# Evaluation of Motor Performances of Hemiplegic Patients Using a Virtual Cycling Wheelchair: An Exploratory Trial

**DOI:** 10.1155/2013/512965

**Published:** 2013-11-26

**Authors:** Norihiro Sugita, Makoto Yoshizawa, Yoshihisa Kojima, Makoto Abe, Noriyasu Homma, Kazunori Seki, Nobuyasu Handa

**Affiliations:** ^1^Graduate School of Engineering, Tohoku University, Sendai, Miyagi 980-8579, Japan; ^2^Cyberscience Center, Tohoku University, Sendai, Miyagi 980-8578, Japan; ^3^Graduate School of Medicine, Tohoku University, Sendai, Miyagi 980-8575, Japan; ^4^Sendai School of Health and Welfare, Sendai, Miyagi 981-3206, Japan

## Abstract

Cycling is known to be an effective rehabilitation exercise for hemiplegic patients who face difficulty during walking because of stroke or other brain disorders. A cycling wheelchair (CWC) is a useful tool to provide exercise for these patients and improve their quality of life. In previous studies, our group developed a system that allows patients to safely practice driving a CWC in a virtual environment. However, it has been difficult to check their motor performances and determine the effects of the exercise on a daily basis. This study is an exploratory trial for developing a method to evaluate the motor performances of users based on their CWC pedaling patterns. An experiment with some hemiplegic patients and healthy subjects was conducted and their pedaling patterns were analyzed. Results showed a significant difference between the hemiplegic patients and healthy subjects in an index that reflects pedaling balance between the feet. This result indicates a possible method of evaluating the motor performances of users based on their pedaling patterns.

## 1. Introduction

Paralysis of the lower extremities can be caused by brain disorders (stroke, brain damage, and degenerative disease), neurologic diseases, or spinal cord injury. In general, patients with severe impairment of the leg use wheelchairs to move around in their daily lives. These patients generally never use their feet while moving with wheelchairs because most wheelchairs are made to be operated either by the user's hands or an electric motor. However, human legs act as a pump for carrying blood back to the heart [1]. Therefore, movement of the feet caused by the calves and the legs of patients does not occur for a long period of time, blood circulation in their lower limbs is reduced, and the risk of disuse syndromes such as muscle weakness and joint contractures is increased.

A cycling wheelchair (CWC) [2–5] is attracting attention as a tool to solve this problem because it provides physical exercise for hemiplegic patients. These individuals can move around more quickly and travel longer distances without fatigue using a CWC instead of a traditional wheelchair. In addition, they can also use their hands while driving a CWC. This encourages the patients to be independent and improves their quality of life. And some previous studies have reported the effectiveness of cycling for hemiplegic patients to improve their motor functions [6–10]. These findings support the possibility that a CWC is useful not only as a transportation device but also as a rehabilitation system for these patients.

Feedback regarding the training effects on patients is important to maintain their motivation for the rehabilitation. In previous studies, existing indices such as the Fugl-Meyer scale [11], the postural assessment scale for stroke patients (PASS) [12], and step length during treadmill use were used to show the effects of cycling. However, specialized instruments and medical staff are required to obtain these indices; therefore, it is difficult for patients to check their motor performances on a daily basis. Furthermore, it is impossible to obtain information on the exercise on a real-time basis.

In preceding studies, we developed a virtual reality (VR) system, in which patients with motor dysfunction can safely drive a CWC without requiring a large open space to practice [13, 14]. This system allows us to measure user movements, such as pedaling, with a high degree of accuracy under several physical conditions. If motor performances and functions of the users are estimated using only their pedaling patterns that are characterized as changes in angular velocity or acceleration of the pedal, it is possible to develop not only a household testing system that checks the motor performances of the users on a daily basis but also new types of rehabilitation systems, for example, a system that automatically selects or plans a rehabilitation program suitable for each user based on their motor performances. In addition, it will be possible to automatically collect a large amount of pedaling data from several patients and to utilize the data for developing effective rehabilitation training methods. The indices obtained in real time will be useful for the development of a rehabilitation system with a biofeedback mechanism [15–18].

The purpose of this study is to develop a new method for assessing the motor performances of users by analyzing their CWC pedaling patterns. In particular, we have proposed new indices that are calculated based on the distribution of angular velocity of CWC pedals. We conducted an experiment to measure the angular velocity of CWC pedals from groups of patients and healthy subjects and discussed the differences in the indices between these groups.

## 2. Methods

### 2.1. Measurement of Pedal Angle

**Figure 1:**
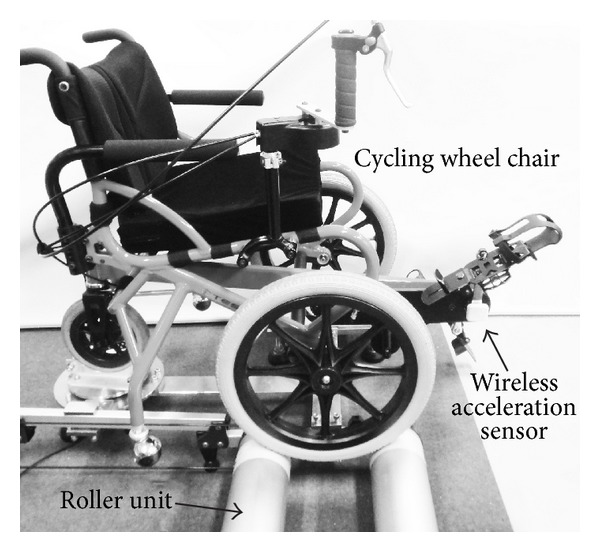
A cycling wheelchair (CWC) fixed to a roller unit of a virtual reality system and a wireless acceleration sensor to measure the angle of the pedal.

**Figure 2:**
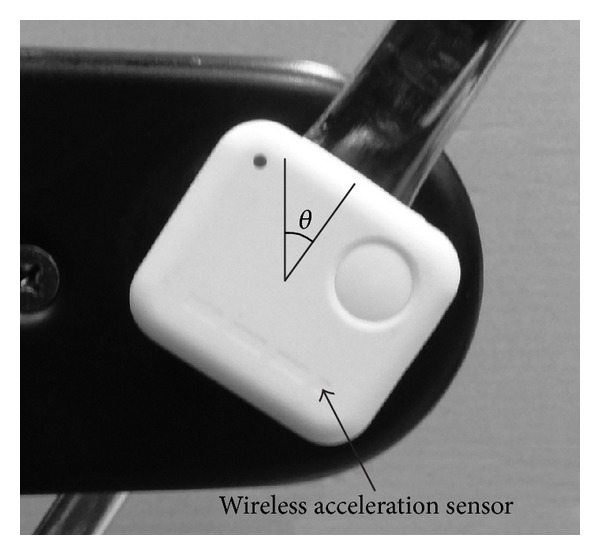
The pedal angle of CWC *θ* (rad) estimated on the basis of the inclination of a wireless acceleration sensor. The pedal angle *θ* is zero when the right pedal is the highest point in a full revolution.

Figure 1 shows the experiment device used in this study. A CWC was fixed to a roller unit of the VR system developed in the preceding study [14]. The minimum torque required to rotate the pedal of the CWC was approximately 5.8 Nm in case of a user weighing 70 kg. Signals from a wireless acceleration sensor (WAA-006, Wireless Technologies, Inc.) attached to the wheel axis were recorded every 0.01 s and filtered through a low-pass filter with a cut-off frequency of 3 Hz. The pedal angle of the CWC *θ* (rad) was estimated on the basis of the inclination of the acceleration sensor as shown in Figure 2. The angle *θ* is defined as zero when the right pedal is at the highest point in a full revolution. The angular velocity of the pedal *ω* (rad/s) was calculated as the difference *θ* in the values.

### 2.2. Analysis

**Figure 3:**
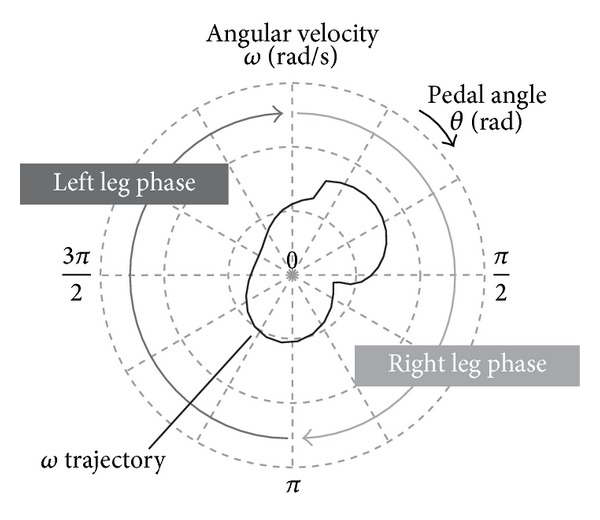
A cobweb chart to illustrate the distribution of the angular velocity in one revolution of the pedal. Right leg phase (0 ≤ *θ* < *π*) and left leg phase (*π* ≤ *θ* < 2*π*) are angular ranges in which the pedal is rotated primary with right or left leg, respectively.

The distribution of the angular velocity *ω* in one revolution of a CWC pedal is graphically illustrated in a cobweb chart (Figure 3). In this figure, the *θ*-axis is set on the circumference of the cobweb chart, and the *ω*-axis is set as an axis perpendicular to the *θ*-axis. In general, healthy persons rotate the pedal primarily with their right leg, when *θ* is from 0 to *π* radian, hereinafter called right leg phase, and they rotate the pedal with their left leg when *θ* is from *π* to 2*π* radian, hereinafter called left leg phase.

**Figure 4:**
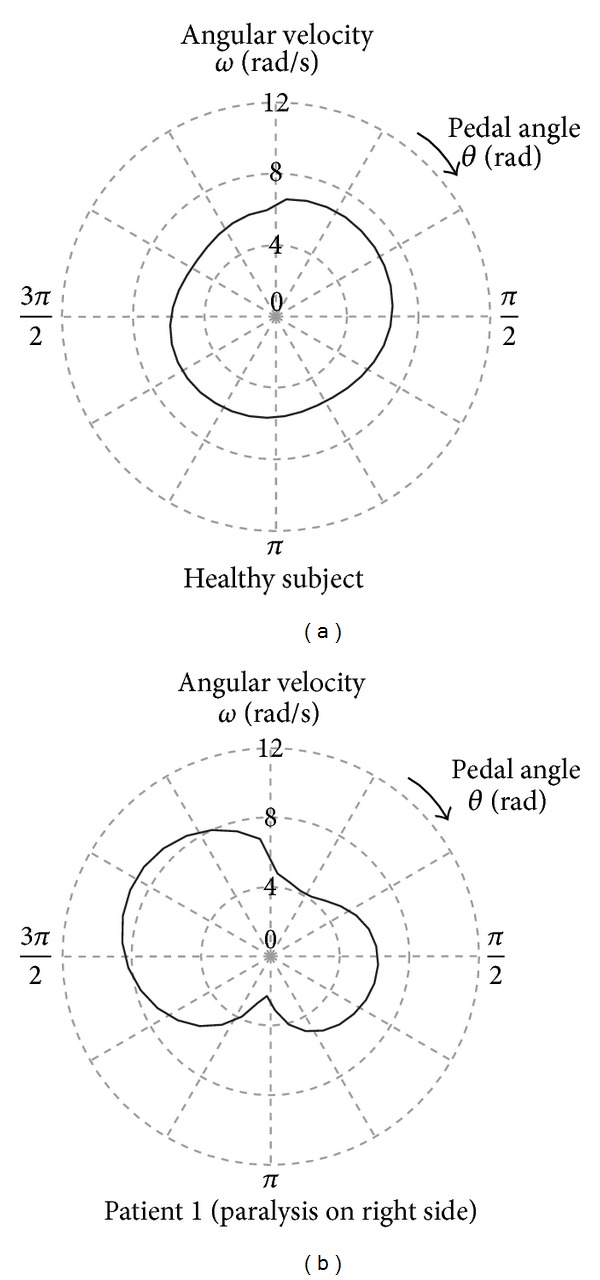
Examples of the angular velocity distribution of CWC pedals in one revolution obtained from (a) a healthy subject and (b) a hemiplegic patient.

**Figure 5:**
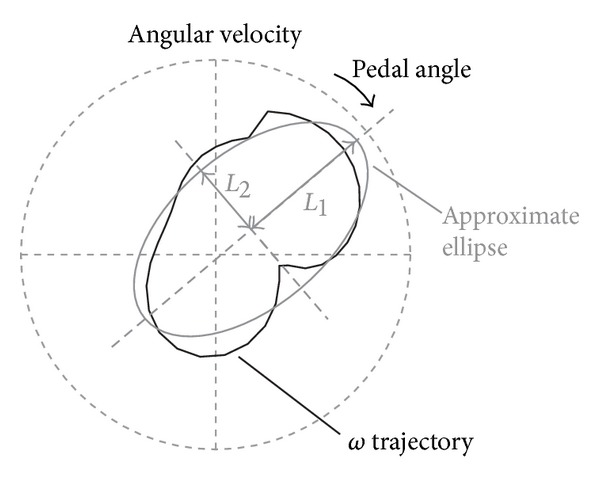
Elliptical approximation of the angular velocity distribution of CWC pedals in a full revolution. *L*_1_ and *L*_2_ are major and minor axes of the approximate ellipse, respectively.

Figure 4 shows examples of the *ω* distributions obtained from (a) a healthy subject and (b) a hemiplegic patient driving the virtual CWC. The patient was paralyzed on his right side because of a stroke. As shown in Figure 4(a), *ω* of the healthy subject was kept relatively constant in one revolution. In contrast, for the patient, there was a difference in *ω* between the right and left leg phases as shown in Figure 4(b). Therefore, the shape of the *ω* distribution obtained from the patient tended to be an ellipse, while that from the healthy subject tended to be a true circle. Based on this observation, a new index, *P*_*b*_, was proposed to evaluate pedaling balance between the feet. *P*_*b*_ is defined as follows:

(1) $$\begin{array}{c} P_{b}=\frac{L_{2}}{L_{1}}, \end{array}$$

where *L*_1_ and *L*_2_ are the major and the minor axes of the approximate ellipse shown in Figure 5, respectively. *P*_*b*_ takes a value from 0 to 1, and the closer to 1 the *P*_*b*_ value, the better the pedaling balance. The ellipse that geometrically approximates the *ω* distribution is obtained by the method of least squares.

Users who are not hemiplegic patients but have some types of disability in their lower body cannot rotate the pedal smoothly during both the right and left leg phases. In this case, the variation in *ω* obtained will be large because the pressure exerted on the pedals is unstable. In this study, pedaling stability was evaluated by calculating an index *P*_*s*_ that is defined as follows:

(2) $$\begin{array}{c} P_{s}=\frac{C}{C_{a}}, \end{array}$$

where *C* and *C*_*a*_ are the path length of the *ω* trajectory and the circumferential length of the approximate ellipse shown in Figure 5, respectively. *P*_*s*_ takes a value from 0 to 1, and the closer to 1 the *P*_*s*_ value, the more stable the rotation of pedaling.

## 3. Experiment

An experiment was conducted to evaluate the effectiveness of the proposed indices. Four hemiplegic patients (4 males; aged 65–75 years; mean age, 68.3 years) and 15 elderly healthy subjects (15 males; aged 61–77 years; mean age, 69.9 years) participated in the experiment. Two out of 4 patients were paralyzed on their right side, and the other 2 patients were paralyzed on their left side because of a stroke. None of the subjects had driven a CWC before this experiment.

A large-screen display was set in front of the experimental system shown in Figure 1 to display a VR image. The subjects sat on the CWC fixed to the system and rotated the pedals more than 10 revolutions while viewing the display. The image on display was updated according to the pedal rotation in real time. They could, therefore, experience a feeling of moving forward. They were instructed to rotate the pedals at a constant rate. But set values of the rate were not provided for them so that they can rotate the pedals in a natural state. The experiment was conducted twice, and data for the analysis was obtained from the second test.

The experimental protocol was approved by the Internal Review Board of the Tohoku University, and informed consent was obtained from all the subjects before the experiment.

## 4. Results

Table 1 shows mean values of *ω*, *P*_*b*_, and *P*_*s*_ in the patient and the healthy subject groups. Each subject's *ω*, *P*_*b*_, and *P*_*s*_ values were calculated on the basis of the data from the 2nd to 7th revolution of the pedals. No significant difference was observed in *ω* and *P*_*s*_ between the two groups in our study. In contrast, *P*_*b*_ of the patients was significantly smaller than that of the healthy subjects (*P* < 0.05; Mann-Whitney *U*-test).

**Figure 6:**
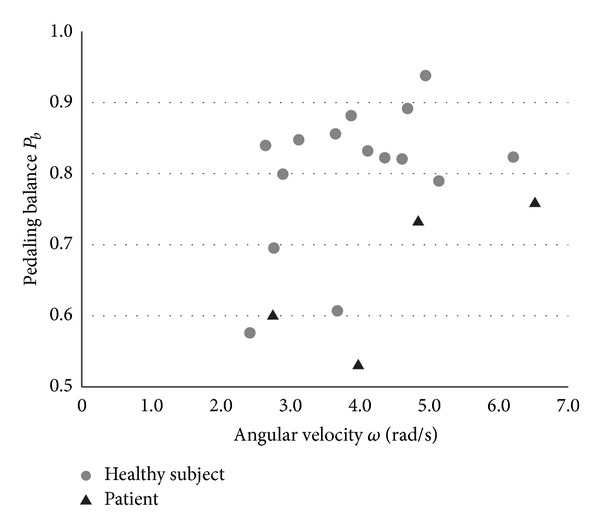
Correlation between angular velocity *ω* and pedaling balance *P*_*b*_. Each subject's data were obtained as a value averaged from the 2nd to 7th revolution of the pedals.

Figure 6 shows the correlation between *ω* and *P*_*b*_. This figure shows that *P*_*b*_ of the patients was lower than that of the healthy subjects, regardless of *ω*. The *P*_*b*_ value was higher than 0.7 for several subjects including the patients; however, *P*_*b*_ obtained from the subjects rotating the pedals at a rate less than 4.0 rad/s decreased even in the healthy group.

**Figure 7:**
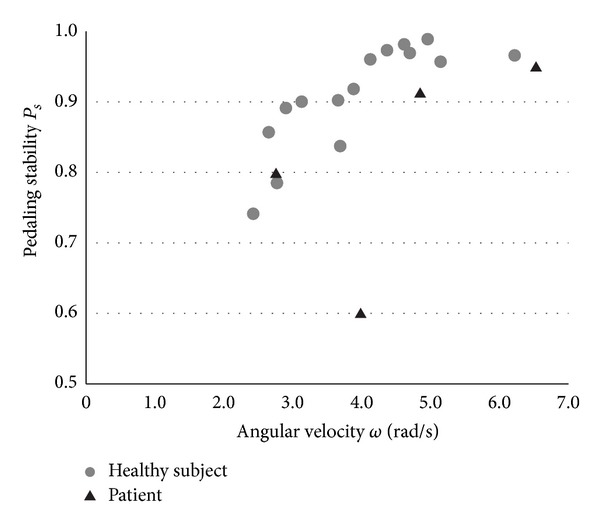
Correlation between angular velocity *ω* and pedaling stability *P*_*s*_. Each subject's data were obtained as a value averaged from the 2nd to 7th revolution of the pedals.

Figure 7 shows the correlation between *ω* and *P*_*s*_. This result shows that the *P*_*s*_ value was higher than 0.7, except in 1 patient, and that *P*_*s*_ was proportional to *ω*. As is the case in *P*_*b*_, *P*_*s*_ values of the patients were lower than those of the healthy subjects when they were compared without considering *ω*.

## 5. Discussion

There was no significant difference in the angular velocity *ω* between the patients and healthy subjects. This result may be related to the rotational load of the roller unit used in the experiment. That is, the rotational load was light enough, so that the patients could rotate the pedals at a high rate using only their unaffected leg.

In contrast, a significant difference was observed in the proposed index *P*_*b*_ that reflects the pedaling balance between the feet. This result indicates the possibility of *P*_*b*_ as an index that can identify hemiplegia symptoms. However, as shown in Figure 6, there were cases in which *P*_*b*_ of healthy subjects rotating the pedals at a low rate decreased considerably. This result implies that maintaining the pedaling balance between the feet at a low rotational rate is difficult even for healthy subjects, and *P*_*b*_ should be evaluated for users rotating the pedals at an adequate rate. From the results of this experiment, the minimum rotational rate desirable for the accurate evaluation of *P*_*b*_ is considered to be 4.0 rad/s.

**Figure 8:**
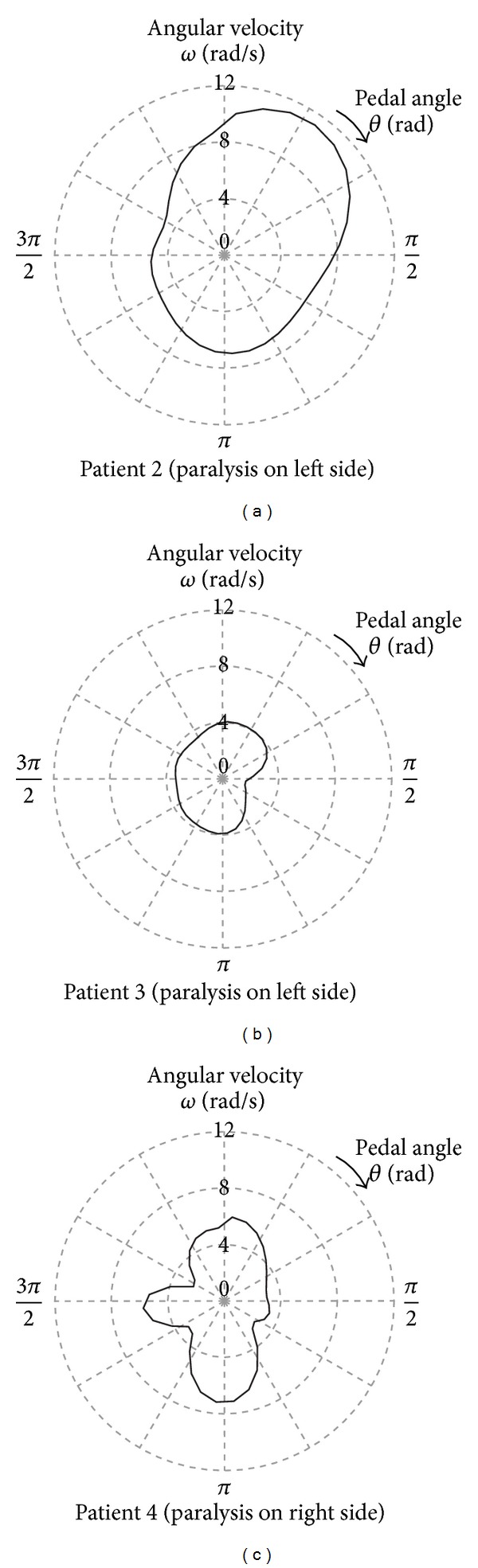
Examples of the angular velocity distribution of CWC pedals in one revolution obtained from (a) and (b) patients with left-sided paralysis and (c) a patient with right-sided paralysis.

Figure 8 shows examples of the *ω* distributions obtained from 3 patients. As shown in Figure 4(b) and Figure 8(a), the patients maintained pedal rotations by raising the rotational speed during the nonparalyze phase, which was the time period when they moved the pedals primarily with their unaffected leg. At the same time, there was a patient who increased the rotational speed during the paralyze phase as shown in Figure 8(b). This patient may have used the strength of his unaffected leg to lift the opposite pedal, on which he propped his affected leg, during the nonparalyze phase, and subsequently the pedal was gravitationally rotated during the paralyze phase. These results indicate that hemiplegic patients have different methods of rotating the pedals.

As shown in Figure 7, the proposed index *P*_*s*_ that reflects pedaling stability was proportional to *ω*. This result is reasonable because the pedal rotation tends to be more stable by the action of the rotational inertia as the rotational rate increases.  Figure 8(c) shows the *ω* distribution of the patient whose *P*_*s*_ was the lowest of all the subjects. This result shows that *ω* changed greatly, even during the nonparalyze phase. In particular, *ω* suddenly increased at approximately the middle point of the nonparalyze phase, approximately *θ* = 3*π*/2, which was the time when the joint angle of the affected leg was the smallest in a full revolution. Therefore, *P*_*s*_ is considered to have a relationship with the range of joint motion.

A center point or a rotational angle of the approximate ellipse of the *ω* distribution may also be useful indices to analyze pedaling patterns. For example, the center point of the approximate ellipse obtained from a patient with right-sided paralysis is considered to be in the left side of the cobweb chart. Furthermore, *P*_*b*_ and *P*_*s*_ can be obtained using a rotary torque or an angular acceleration of the pedal instead of the angular velocity. These indices may provide detailed information of pedaling characteristics in an easy-to-understand way because they are closely correlated with muscle activation patterns of users. However, a torque sensor requires major reorganization of the CWC, and the angular acceleration is easily influenced by noises such as wheel wobble; thus a higher-accuracy method for measuring the pedal angle is required.

The wireless acceleration sensor used in this study is easy to attach and is indestructible. It is, therefore, suitable for household systems to check the motor performances of users as noted in the Introduction. However, accuracy of the pedal angle obtained with the acceleration sensor is not considerably high compared with that with mechanical sensors such as a rotary encoder. Some noises, such as the centrifugal force and vibrations of the CWC, reduce the accuracy of the pedal angle. Estimation errors caused by these factors may be reduced by combining different types of sensors.

## 6. Conclusion

In this study, we analyzed pedaling patterns of the angular velocity obtained from hemiplegic patients and healthy subjects driving a CWC in a virtual environment. Two new indices based on the angular velocity of the CWC pedals were proposed to evaluate the motor performances of users.

The experimental results showed that a significant difference between the hemiplegic patients and healthy subjects was observed in the index related to the pedaling balance of the users between their feet. This result indicates a possibility of evaluating motor performances and functions of CWC users based on their pedaling patterns. In contrast, it was shown that accuracy of this index decreased for subjects rotating the pedals at a low rate.

In future works, changes to the proposed indices before and after rehabilitation trainings should be analyzed to test their efficiency. In addition, it is necessary to compare the proposed indices with general and traditional ones that are known to reflect the degree of motor dysfunctions [11, 12]. To investigate the relationship between the symptomatic states of users and pedaling patterns, more patient data are required. In addition, influences of rotational speed on the proposed indices should be removed as much as possible. A rotational rate appropriate for pedaling differs between the patient and healthy subject groups; therefore, a new index that is insusceptible to the rotational rate is required. Rotational loads of the CWC are considered to have significant influence on the pedaling patterns. A detailed analysis will be possible if data are obtained under conditions of some different rotational loads.

## Figures and Tables

**Table 1:** Comparison of mean *ω*, *P*_*b*_, and *P*_*s*_ values between the patient and healthy subject groups.

	Patient (*n* = 4)	Healthy subject (*n* = 15)
Angular velocity (*ω*)	4.52 ± 1.59 rad/s	3.94 ± 1.07 rad/s
Pedaling balance (*P*_*b*_)	0.66 ± 0.10*	0.80 ± 0.10*
Pedaling stability (*P*_*s*_)	0.81 ± 0.16	0.91 ± 0.075

*P < 0.05; Mann-Whitney *U*-test.
